# CT Evaluation of Lumbar Interbody Fusion: A Comprehensive Review with an Integrated Framework for Principle-Based Interpretation

**DOI:** 10.3390/diagnostics16010140

**Published:** 2026-01-01

**Authors:** Szu-Hsiang Peng, Jwo-Luen Pao

**Affiliations:** 1 Department of Medical Imaging, Far Eastern Memorial Hospital, New Taipei City 22060, Taiwan; deer530530@gmail.com; 2 Department of Orthopedic Surgery, Far Eastern Memorial Hospital, New Taipei City 22060, Taiwan

**Keywords:** computed tomography, lumbar interbody fusion, pseudarthrosis, trabecular bridging, metal artifact reduction, Brantigan-Steffee-Fraser classification, zone-based assessment, principle-based interpretation

## Abstract

**Background/Objectives:** Computed tomography remains the reference standard for assessing lumbar interbody fusion, yet significant methodological heterogeneity, documented across more than 250 different assessment combinations, directly impacts treatment decisions and outcome reporting. The main challenge is applying uniform criteria to technique-specific anatomical configurations that generate distinct bridging patterns. **Methods:** This narrative review synthesizes evidence from 2000 to 2025 through PubMed and Google Scholar searches, examining imaging protocols, radiographic criteria validated against surgical exploration and reliability studies, and classification systems with emphasis on clinical application. **Results:** Modern protocols that incorporate iterative metal artifact reduction and dual-energy imaging substantially improve visualization of the hardware–bone interface. Zone-based evaluation shows that bridging patterns primarily reflect cage configuration and graft placement strategy rather than the surgical approach alone—a key distinction that affects assessment methodology. Validation studies confirm higher inter-observer reliability for extracage zones (ICC 0.79–0.84) compared to intracage regions (ICC 0.70–0.79). Evidence supports three main bridging patterns: graft-dependent consolidation, ungrafted-zone bridging, and accessibility-dependent variation. Assessment at 12 months captures most successful fusions, although 15–16% show delayed progress and require longer follow-up. **Conclusions:** This review synthesizes current evidence on technical optimization and temporal healing patterns, proposing a principle-based interpretive framework that accommodates technique-specific differences instead of strict categorical criteria. This framework allows personalized assessment correlated with surgical documentation, addressing the documented heterogeneity while enhancing diagnostic consistency.

## 1. Introduction

Lumbar interbody fusion (LIF) is one of the most common spinal procedures, usually performed for degenerative disc disease, spondylolisthesis, or spinal instability. By 2015, nearly 200,000 procedures were performed annually in the United States, costing over $10 billion [1,2]. Pseudarthrosis remains a significant complication, with reported rates ranging from 7% to 20%, depending on the surgical approach and patient factors [2]. The risk is substantially higher in multi-level procedures and in patients with osteoporosis, with rates reaching up to 29% even in single-level fusion [2]. Failed fusion can lead to ongoing pain, hardware-related complications, and the need for revision surgery. Therefore, accurate radiographic assessment is crucial for guiding postoperative management [1,2].

Computed tomography (CT) has become the preferred imaging modality for assessing interbody fusion, as reflected in current clinical practice guidelines [3]. CT provides superior visualization of trabecular bone bridging through high-resolution multiplanar reconstruction compared to plain radiography, which has limited ability to evaluate osseous integration within fusion cages. Radiolucent cage materials help visualize bone grafts by minimizing obscuration of the fusion bed, while metallic implants may conceal trabecular bridging patterns [3,4]. Modern iterative reconstruction algorithms and dual-energy imaging further improve hardware–bone interface visualization by substantially reducing metal artifacts [3,5], establishing thin-section CT with multiplanar reconstruction as the current reference standard [3,5]. Validation studies comparing surgical exploration show varying diagnostic accuracy depending on different fusion criteria and cage materials, with sensitivity ranging from 20% to 93% and specificity from 46% to 92% [4,6]. Nonetheless, meta-analyses comparing imaging modalities with surgical exploration confirm that CT has superior diagnostic accuracy over plain radiography, flexion-extension radiography, and scintigraphy [7].

Despite these advances, assessing fusion across studies remains challenging. Recent systematic reviews analyzing over 400 lumbar interbody fusion studies documented significant methodological heterogeneity: 18 different fusion definitions and more than 250 unique assessment method combinations, with 45% of these combinations appearing in only one study [6,8]. The clinical impact is significant. For example, fusion rate can range from 74% to 100%, depending solely on a single assessment criterion used [6]. This proliferation of assessment methods happens despite extensive use of descriptive criteria and formal classification systems [6]. Heterogeneity of assessment methods highlights three key challenges. Diversification of surgical approaches—especially the widespread adoption of lateral techniques such as lateral lumbar interbody fusion (LLIF) and oblique lumbar interbody fusion OLIF) after 2015 [9,10]—has created distinct anatomic corridors and cage configurations that generate technique-dependent bridging patterns inadequately addressed by traditional classification systems [11,12]. Inter-observer variability remains when using different classification systems, with agreement levels ranging from poor to fair (κ = 0.20–0.35 for categorical grading systems) [6], particularly for equivocal bridging patterns. Additionally, the biological continuum of osseous healing resists strict categorization, as shown by “locked pseudarthrosis”, which demonstrates biomechanical stability without complete trabecular bridging [13]. This fundamental disconnect between radiographic appearance and clinical status, with outcome correlations as low as r < 0.26 [14], further complicates interpretation of assessments, as anatomic bridging does not reliably predict symptom resolution or biomechanical stability.

While recent systematic evidence has recommended standardization on CT-based continuity of bony bridging [6], practical implementation requires interpretive frameworks that accommodate documented technique-specific variation without enforcing rigid approach-based criteria for each surgical permutation.

Given these challenges, this narrative review offers a framework for contemporary fusion assessment. We synthesize evidence on CT imaging protocols optimized for current instrumentation and radiographic criteria—including trabecular bridging evaluation, zone-specific patterns, secondary signs, and documentation standards. The review highlights a principle-based interpretation that considers technique-specific factors: cage configuration, graft placement, and surgical accessibility. This framework correlates radiographic findings with surgical documentation and temporal healing patterns, addressing the documented heterogeneity in fusion assessment.

## 2. Materials and Methods

This narrative review synthesizes current evidence on CT-based evaluation of lumbar interbody fusion.

A comprehensive search of PubMed and Google Scholar was conducted for English-language studies published between 2000 and 2025, using combinations of the terms ‘lumbar fusion,’ ‘interbody fusion,’ ‘ALIF,’ ‘TLIF,’ ‘LLIF,’ ‘OLIF,’ ‘computed tomography,’ ‘CT assessment,’ ‘fusion criteria,’ ‘pseudarthrosis,’ and ‘classification systems.’ Reference lists of key articles were also reviewed to identify additional relevant publications.

Articles were included if they addressed CT imaging protocols, radiographic fusion criteria, classification systems, inter-observer reliability studies, or validation studies comparing CT with surgical exploration. Priority was given to prospective studies, systematic reviews, and multicenter validations. Studies published after 2015 were prioritized for evaluation of modern artifact-reduction techniques and lateral approaches. Studies were excluded if they focused exclusively on posterolateral fusion, addressed non-CT modalities in isolation, or reported small case series (<20 patients) evaluating fusion outcomes without validation data. Technical or methodological studies were included, regardless of sample size, if they provided relevant validation of imaging techniques.

Content was organized thematically into CT imaging protocols, radiographic assessment criteria, and documentation standards.

## 3. Results

CT-based fusion assessment integrates technical optimization with systematic radiographic evaluation. Section 3.1 provides evidence-based protocols for CT acquisition, reconstruction, artifact management, and assessment timing. Section 3.2 defines comprehensive assessment criteria covering trabecular bridging patterns, zone-specific evaluation principles, secondary radiographic signs, and standardized documentation frameworks. The clinical integration of these technical and radiographic components is outlined in Section 4.3.

### 3.1. CT Imaging Protocols

CT is the primary imaging modality for postoperative fusion assessment, providing superior osseous detail and detecting trabecular bridging earlier than radiography [3,5]. High-spatial-resolution imaging with multiplanar reformations enables thorough assessment of bone bridging, hardware integrity, and complications [3,5]. However, the protocol needs to be optimized to minimize artifacts produced by metallic implants and ensure image quality.

#### 3.1.1. Acquisition Parameters

Optimal CT protocols for postoperative fusion evaluation require balancing among spatial resolution, radiation dose, and metal artifact reduction. Using high tube voltage (120–140 kilovolt peak (kVp)), along with adjusted tube current and pitch, is crucial for penetrating metal implants and minimizing beam-hardening artifacts [5,15]. Thin-section acquisition enhances visualization of trabecular bridging. Modern protocols usually use slice thicknesses of 1–3 mm, balancing spatial resolution with manageable noise levels and radiation exposure [3,5]. Multiplanar reformations in coronal and sagittal planes help evaluate osseous continuity across the fusion construct [3,5].

#### 3.1.2. Hardware-Specific Considerations

Hardware composition greatly impacts artifact severity. Titanium alloy produces fewer artifacts than stainless steel, enabling clearer visualization of surrounding bone structures [5,15]. PEEK (polyetheretherketone) has radiolucent properties, resulting in minimal artifacts on CT [5], which enhances visualization of intracage bone formation and the cage-endplate interface compared to metallic cages [15].

Despite optimal cage selection, residual artifacts from posterior fixation hardware—pedicle screws and rods—remain the main source of imaging degradation in modern instrumented fusion. Posterior instrumentation spans multiple vertebral levels, producing beam-hardening artifacts that may obscure nearby extracage zones. The extent of the artifact burden determines whether direct trabecular visualization is feasible or whether assessment must rely on indirect signs and artifact-reduction techniques.

#### 3.1.3. Artifact Reduction Strategies

Artifact management starts with proper reconstruction parameters. Using soft-tissue algorithms and extended Hounsfield scales reduces artifacts from metal hardware while preserving bone detail [3,5]. Modern CT systems also incorporate advanced artifact reduction technologies that greatly improve the assessment of instrumented spines.

- Iterative Metal Artifact Reduction (IMAR)

**Figure 1:**
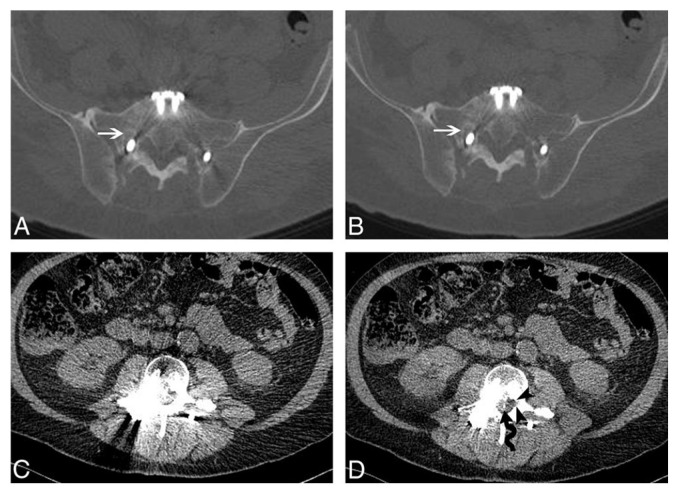
Filtered back projection (FBP) versus iterative metal artifact reduction (IMAR) in a 65-year-old man after L3–S1 instrumentation. At S1 (upper row, bone window), both FBP (**A**) and IMAR (**B**) demonstrate pedicle screw lucency consistent with loosening (white arrows). At L3 (lower row, soft-tissue window), FBP (**C**) shows severe artifact obscuring the central canal, while IMAR (**D**) reveals improved visualization of the lateral recess (black arrowheads) and a retained spinal cord stimulator wire (black wavy arrow). (Reprinted with permission from reference [16]).

Conventional CT reconstruction using filtered back projection (FBP) is susceptible to beam-hardening artifacts caused by metallic hardware. IMAR algorithms address this issue through projection-based correction, significantly enhancing the visualization of soft-tissue structures near hardware, with improvements observed in 88% of patients [16] (Figure 1).

IMAR algorithms can, however, create secondary artifacts that mimic pathology. A systematic review of 899 studies found secondary artifacts in up to 82% of spine imaging cases [17]. Artifactual lucency around pedicle screws may mimic hardware loosening; hypodense artifacts in the spinal canal could be mistaken for arachnoiditis or layering debris; or irregular hyperdense lesions might be confused with bone cement fragmentation. When such artifactual lucency is suspected, comparison with non-IMAR (standard) reconstructions is essential to verify the true hardware–bone interface before diagnosing loosening [18].

- Dual-Energy CT (DECT)

**Figure 2:**
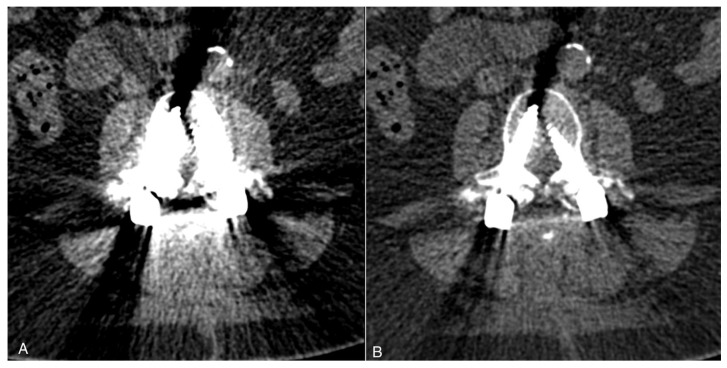
Effect of keV selection on metal artifact reduction in dual-energy CT. Axial images at L3 level in a 62-year-old woman with posterior instrumentation (L3–S1 pedicle screws and rods). (**A**) At 100 keV, a significant beam-hardening artifact obscures the hardware–bone interface. (**B**) At 140 keV, artifact burden is substantially reduced, improving visualization of pedicle screw-bone interfaces. (Reprinted with permission from reference [5].).

DECT reduces beam-hardening artifacts through virtual monoenergetic reconstruction. High-keV reconstructions (120–140 kiloelectron volt (keV)) significantly enhance visualization of the hardware–bone interface [19] (Figure 2).

- Combined Approach

Combining iterative reconstruction and monochromatic imaging provides synergistic artifact reduction while preserving image quality, a combination increasingly recommended in modern protocols to optimize fusion assessment and hardware integrity evaluation [20].

#### 3.1.4. Patient Positioning

Standard supine positioning with image acquisition perpendicular to the implant is recommended for routine fusion evaluation [3,5,15]. This method decreases artifact severity and supports consistent evaluation across serial exams.

#### 3.1.5. Timing of CT Assessment

CT demonstrates superior sensitivity in detecting early trabecular bridging, with initial bone formation visible by 3 months after surgery. Signs of trabecular bridging around or through interbody grafts usually develop into mature trabecular bridging between 6 and 12 months [3,5].

Large-scale longitudinal data show that most successful fusions consolidate within 12 months [21]. However, 15–16% of ultimately successful fusions show incomplete bridging at 12 months but then progress to solid arthrodesis by 17 months, indicating that radiographic maturation may lag behind mechanical stability [21,22]. Fusion progression beyond 17 months without evidence of bridging becomes less likely [21].

These temporal patterns show considerable variability in individual healing paths. Incomplete bridging observed at 3–6 months is typical of normal healing, with about two-thirds of early peri-implant lucency resolving spontaneously without intervention [21]. This biological timeline informs evidence-based assessment protocols (Section 4.3.1).

### 3.2. Radiographic Assessment Criteria

Trabecular bridging across the interbody space is the primary criterion for achieving solid fusion [3,4,23], representing the most consistently used assessment parameter in the literature (used in 89% of lumbar interbody fusion studies) [6]. Modern artifact-reduction techniques improve the accuracy of assessing bridging bone in instrumented spines [16]. A comprehensive fusion assessment involves systematically evaluating bridging patterns, bridging zone-specific features, and secondary signs.

#### 3.2.1. Trabecular Bridging: The Primary Criterion

The minimum extent of bridging required for solid fusion remains debated, with suggested thresholds ranging from partial bridging to full circumferential osseous continuity [13,23]. Current consensus, based on the Brantigan-Steffee-Fraser (BSF) classification, favors trabecular bone bridging over at least 50% of the fusion area without lucency as an indicator of successful arthrodesis [4,24].

Trabecular bridging occurs in distinct anatomical compartments with distinct diagnostic features. Intracage bridging occurs within cage fenestrations, although metallic artifacts often interfere with direct visualization [3,13]. Extracage bridging forms outside device boundaries; validation studies demonstrate higher rates of extracage bridging among fused segments and greater diagnostic reliability compared with intracage assessment [11,12].

Trabecular bridging patterns are classified by their completeness: absent bridging indicates no trabecular continuity; incomplete bridging shows partial trabecular connection; and complete bridging demonstrates uninterrupted trabecular continuity from endplate to endplate without radiolucent lines [12,25].

#### 3.2.2. Bridging Zone-Based Assessment: Validation Findings

Validation studies reveal that bridging distribution depends on three key technical factors: graft placement strategy, cage geometric configuration, and surgical accessibility. The following subsections outline the methodology for bridging zone-based assessment, followed by empirical evidence that demonstrates how each factor affects bridging patterns across anatomical compartments.

- Assessment Methodology and Reliability Validation

**Figure 3:**
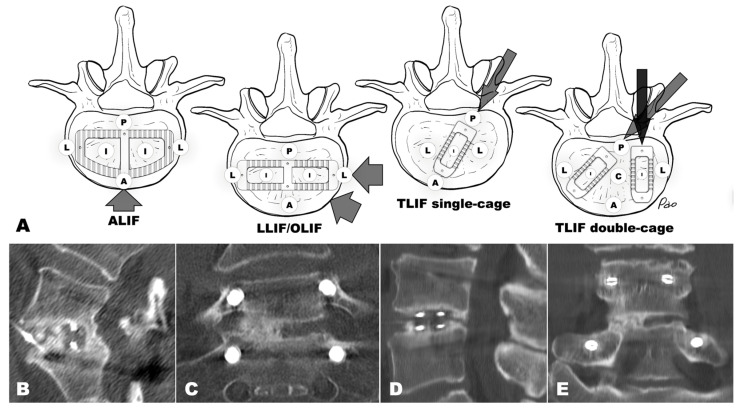
Zone-based assessment of lumbar interbody fusion: anatomic definitions and CT demonstration. (**A**) Schematic illustrations showing the anatomic distribution of bridging zones (A = anterior extracage, P = posterior extracage, L = lateral extracage, I = intracage, C = intermediate extracage) relative to cage positioning in different interbody fusion configurations. Arrows indicate the surgical approaches. LIF, lumbar interbody fusion; ALIF, anterior LIF; LLIF, lateral LIF; OLIF, oblique LIF; TLIF, transforaminal LIF. (**B**–**E**) CT demonstration of complete versus incomplete posterior extracage bridging following biportal endoscopic transforaminal lumbar interbody fusion (BETLIF). (**B**,**C**) Complete bridging at L5/S1, 2-year follow-up: sagittal (**B**) and coronal (**C**) reformations demonstrate continuous trabecular bridging in the posterior extracage zone with endplate-to-endplate bone incorporation. (**D**,**E**) Incomplete bridging at L4/5, 5-year follow-up: sagittal (**D**) and coronal (**E**) reformations show bone formation in the posterior extracage zone without continuous endplate-to-endplate bridging, compatible with a “locked pseudoarthrosis”; hardware remains stable without peri-implant lucency or cage migration.

Bridging zones are defined as anatomical regions relative to interbody devices: anterior (ventral to the cage), posterior (dorsal to the cage), lateral (beside the cage), and intracage (within cage fenestrations). Double-cage configurations include an intermediate zone between the devices [12,26] (Figure 3). Assessment distinguishes absent, incomplete, and complete bridging using multiplanar sagittal and coronal verification [12]. Validation studies show higher inter-observer agreement for extracage zones (intraclass correlation coefficient (ICC) 0.79–0.84) compared to intracage assessment (ICC 0.70–0.79), with posterior extracage zones demonstrating the highest reliability [12]. Importantly, zone-specific reliability and fusion rate data derive exclusively from TLIF cohorts—no other approach has comparable prospective validation [12,26]. ALIF zone-based principles—including posterior ungrafted zone bridging as the most reliable fusion indicator—were established through surgical exploration validation but lack adequately powered inter-observer reliability data [11]. Lateral approach assessment criteria are extrapolated from biomechanical and cage geometry principles without dedicated prospective validation. This evidence hierarchy should inform interpretation when applying zone-based principles across surgical approaches (detailed in Section 4.4.2).

- Graft Placement and Bridging Prevalence

Among the factors influencing bridging distribution, direct bone graft placement is associated with a higher rate of bridging. Grafted zones consistently show higher complete bridging rates than ungrafted zones, although the exact values vary depending on cage configuration and assessment methodology. In double-cage TLIF with anterior extracage grafting, grafted zones (intracage and anterior extracage) show 43–48% complete bridging at 12 months, compared to 31% in the ungrafted posterior extracage zone [12]. In single-cage TLIF, the grafted intracage zone achieves 95% fusion, while the ungrafted posterior extracage zone reaches 74% [26].

Bridging also happens frequently in ungrafted areas at clinically relevant rates. Posterior extracage bridging—forming without direct graft placement—is seen in about 31% of double-cage TLIF [12] and 74% of single-cage configurations [26]. Such bone formation in ungrafted zones is regarded as a reliable sign of successful fusion, as it indicates osteoinduction within the properly stabilized segment [11]. These findings suggest that bridging distribution depends on both graft placement and the biomechanical environment; expected patterns should be understood in the context of documented surgical techniques rather than universal numerical thresholds.

- Cage Geometry Effects

Cage footprint affects the distribution of lateral zone bridging. Large-footprint devices that span the full disc width show bilateral lateral bridging [27]. Shorter cage configurations demonstrate anterior-lateral patterns [27,28]. Correlation between the observed bridging distribution and actual cage dimensions allows for a more detailed assessment based on implant geometry rather than solely on the surgical approach.

- Surgical Accessibility and Bridging Rates

Surgical accessibility correlates with bridging prevalence regardless of graft placement and cage design. Single-cage validation showed 26% fusion prevalence in contralateral zones compared to 67% in ipsilateral zones [26]. The quality of endplate preparation was associated with a 3- to 4-fold variation in endplate cystic changes and cage subsidence rates [29]. These accessibility-related differences suggest that ipsilateral-contralateral bridging variations reflect surgical execution factors that can be linked to operative documentation.

- Assessment Considerations

Bridging zone-based assessment requires distinguishing it from confounding findings such as osteophytes (bony projections without trabecular continuity crossing the disc space) [11,30] and endplate cystic change following endplate injury [29]. Advanced artifact reduction strategies reduce metal artifacts from cage fenestrations and posterior fixation hardware [16,18]. Thin-slice CT protocols with multiplanar reconstruction showed increased diagnostic accuracy for bridging zone-based assessment [29]. Integration of these zone-specific bridging patterns with surgical documentation and temporal healing context guides principle-based interpretation, as detailed in Section 4.3.

#### 3.2.3. Secondary Signs and Complications

Secondary signs support trabecular bridging assessment, offering essential diagnostic details when direct visualization of bridging is limited or uncertain [3,31]. Key secondary signs include cage subsidence, changes at the endplate-cage interface, and hardware integrity [3]. Implant-related complications—especially cage subsidence and migration—require systematic evaluation due to their impact on fusion outcomes [27,31]. New-onset traction spur formation after fusion surgery—horizontal osteophytes appearing 2–3 mm from vertebral margins—may signal persistent instability and pseudarthrosis, although diagnostic specificity is high and sensitivity is limited [30]. Interpreting these findings depends on the timing, as the same radiographic appearances can have different clinical significance based on the postoperative interval.

- Cage Subsidence

Cage subsidence—implant sinking into nearby vertebral endplates—represents an early postoperative phenomenon, with most cases noticeable within the first month and stabilizing by three months, predominantly affecting the inferior endplate [31]. Primary risk factors include osteoporosis and endplate injury during surgical preparation, with patients showing low vertebral Hounsfield unit (HU) values at significantly higher risk [31,32]. Surgical approach also contributes to the prevalence of subsidence, with posterior techniques demonstrating higher rates than anterolateral approaches [33].

Subsidence classification relies on absolute measurements. Disc height loss of 2 mm or less does not meet the threshold for clinically significant subsidence. Mild subsidence (2–4 mm) has minimal impact on fusion outcomes. In contrast, severe subsidence of more than 4 mm indicates notable failure at the cage-endplate interface and requires clinical correlation, as it may affect disc height restoration and indirect neural decompression. OLIF with anterolateral fixation showed lower fusion rates in patients with severe cage subsidence (64.5% vs. 92.6%, *p* < 0.001). Serial CT examination tracks progressive subsidence beyond 3 months, with ongoing progression suggesting inadequate structural support [31]. Quantitative assessment of bone quality using preoperative vertebral HU measurements provides objective risk stratification. Level-specific thresholds (L4: 127 HU, L5: 136 HU, S1: 142 HU) identify patients at increased risk of combined nonunion and subsidence (*p* = 0.022) [32].

- Cage Migration Patterns

The migration assessment evaluates the direction of displacement and anatomical proximity. CT identifies the direction of displacement and anatomical relationships with neural and vascular structures. Posterior or lateral displacement toward the neural foramina may cause nerve root compression. Anterior displacement poses a risk of vascular and visceral injury [27,34]. Clinical significance depends on symptom correlation rather than displacement magnitude. Asymptomatic stable migration may only require observation, whereas displacement causing new neurological symptoms warrants consideration of intervention.

- Endplate-Cage Interface Changes

Endplate cystic changes near fusion devices suggest inadequate mechanical stability and delayed fusion, which are different radiographic findings from structural issues such as cage subsidence. Unlike ongoing hardware failure, these interface changes exhibit dynamic behavior: their resolution on serial imaging indicates progression toward solid arthrodesis. While endplate cystic changes signal delayed fusion, they may resolve as arthrodesis advances; additional fixation can help promote this progression in certain cases [3].

- Hardware Integrity

Key indicators of hardware issues include peri-implant radiolucency (haloing) around fusion devices and pedicle screws, progressive changes in screw orientation suggesting loosening, and hardware fractures indicating mechanical failure [3,5].

Interpretation of peri-implant lucency relies on serial follow-up to track changes over time. Peri-implant lucent zones larger than 2 mm around pedicle screws suggest possible hardware loosening; however, a definitive diagnosis requires demonstrating a change in implant position on serial imaging [3,5]. Notably, early postoperative radiolucent zones do not necessarily mean fusion failure, as about two-thirds of levels with initial radiolucency eventually achieve solid fusion. This lucency typically occurs during early fusion stages and often resolves with successful arthrodesis; on the other hand, new radiolucency after bridging bone formation may indicate a pathological process that needs further assessment [21]. Artifactual lucency caused by metal artifact reduction algorithms must be ruled out through comparison with standard reconstructions before diagnosing true hardware loosening (Section 3.1.3) [18].

Hardware fracture can occur through different mechanisms. Delayed fracture indicates failure due to pseudarthrosis or infection, usually happening after the expected fusion period [15]. However, early hardware complications do not always mean pseudarthrosis, as about 60% of segments with early implant failure still achieve solid fusion when posterior instrumentation remains stable. Longitudinal studies show that all hardware failures happened before bridging bone formed, with no new failures after bridging was established [21]. Key diagnostic thresholds for secondary radiographic signs are summarized in Table 1 for quick reference during CT interpretation; detailed clinical factors regarding timing, risk, and management are discussed throughout the text.

#### 3.2.4. Documentation Framework and Reference Standards

Despite the proliferation of fusion grading systems, the BSF classification remains the most widely validated framework for documenting interbody fusion. BSF classification provides three-tier grading with well-defined radiographic criteria, validated against surgical exploration in multiple cohorts [4,13]:

BSF-1 indicates radiographic pseudarthrosis, characterized by construct collapse, loss of disc height, vertebral slip, cage displacement, or significant bone resorption around the implant, including hardware failure and peri-implant lucency [4].

BSF-2 indicates “locked pseudarthrosis”—a clinically important intermediate condition marked by mid-cage radiolucency despite solid bone growth from each vertebral endplate into the cage periphery [4]. Although radiographically incomplete, this pattern correlates with positive clinical outcomes in many patients [13]. Longitudinal analyses demonstrate that once bridging bone engages both endplates, further deterioration becomes unlikely [21]; however, clinical interpretation in symptomatic patients requires individualized assessment.

BSF-3 indicates radiographic fusion, characterized by trabecular bone bridging at least 50% of the fusion area with bone density similar to that observed immediately after surgery [4], serving as the gold standard for solid arthrodesis.

Validation studies demonstrate high diagnostic accuracy for BSF classification, with overall accuracy exceeding 90% and 100% sensitivity for detecting pseudarthrosis [4], as well as substantial inter-observer agreement (*κ* = 0.77–0.88) in studies using modern titanium fusion cages [35]. Despite technical accuracy, correlation with clinical outcomes remains weak (r < 0.26) [14]. This clinical-radiographic disconnect highlights the need to supplement categorical grading with detailed documentation of bridging location (fusion zone-specific patterns, Section 3.2.2), temporal context (months postoperative, progression on serial imaging), and relevant complications (subsidence, hardware integrity). Combining these radiographic findings with clinical presentation and surgical documentation enables individualized interpretation, as explained in Section 4.3.

## 4. Discussion

### 4.1. Literature Context and Review Contribution

Given the practical orientation of this narrative review, Section 4.2 and Section 4.3 intentionally integrate evidence synthesis with clinical decision-making guidance. This structure departs from the typical discussion format to provide radiologists and spine surgeons with immediately practical assessment frameworks.

Previous reviews established fundamental CT assessment principles, with Williams et al. supporting thin-section protocols and multiplanar reconstruction [3]. However, these guidelines predate modern artifact-reduction techniques and the subsequent diversification of surgical approaches [9,16,19,36].

This review advances the field by synthesizing post-2015 technical developments with bridging zone-based validation evidence to propose a principle-based interpretive framework [11,12,26]. Unlike prior approaches that seek universal categorical criteria, this framework accommodates documented technique-specific variation by systematically correlating with surgical documentation and temporal healing patterns—addressing the substantial methodological heterogeneity documented in contemporary literature [6,8].

### 4.2. Integration of Current Evidence

#### 4.2.1. Technical Optimization and Zone-Based Validation

Contemporary CT protocols provide a robust technical foundation supporting principle-based fusion assessment. When optimal visualization is achieved using radiolucent cages, CT shows 100% sensitivity for detecting pseudarthrosis, as validated by surgical exploration [4]; however, sensitivity reported across studies varies widely from 20% to 93% depending on the cage materials and assessment criteria [6], reflecting how metallic artifacts affect evaluation. Artifact-reduction techniques, such as IMAR and dual-energy CT, help mitigate this issue by enhancing visualization in the vicinity of instrumentation [16,19]. Using thin-cut protocols with multiplanar reconstruction allows for systematic, bridging zone-specific evaluation [11,12].

Zone-based validation identifies three empirically distinct bridging patterns. Graft-dependent consolidation is most common in zones receiving direct osteogenic material. Ungrafted-zone bridging occurs at notable rates, challenging simple models that equate graft presence with fusion potential. Accessibility-dependent patterns show significant differences between technically challenging and easily accessible zones, indicating that the quality of technical execution influences outcomes regardless of graft placement [11,12,26].

The coexistence of these patterns reveals that fusion is not a uniform process controlled solely by the surgical approach. The notable bridging capacity observed in ungrafted zones and the apparent variation with accessibility, as documented in Section 3.2.2, indicate that cage configuration, graft placement strategy, and surgical execution together influence the distribution of bridging [12,26]. Cage geometry also affects expected bridging patterns, with footprint dimensions impacting lateral zone bridging distribution [27,28].

These findings demonstrate that bridging distribution involves multiple interacting factors beyond just surgical approach categories alone. Systematic interpretation of these patterns requires frameworks that account for documented variation, as discussed in subsequent sections.

#### 4.2.2. Challenges to Categorical Assessment Frameworks

The empirical patterns described in Section 4.2.1 support the assessment challenges outlined in the Introduction, showing why rigid categorical frameworks are inadequate for modern practice.

Regarding technique-specific variation, the 2.5-fold difference between accessible (67%) and challenging zones (26%), distinct bridging distributions between double-cage and single-cage configurations, and between fusion cages of different footprint sizes, confirms that universal criteria cannot accommodate the documented technical heterogeneity [12,26,27,28]. This complexity contributes to the poor inter-observer agreement noted in the Introduction. However, reliability improves substantially when standardized protocols are applied to modern titanium constructs (Section 3.2.4) [35].

Regarding temporal complexity, the delayed fusion phenomenon documented in Section 3.1.5 confirms that categorical systems assigning definitive status at single time points inadequately represent the biological healing continuum.

Regarding the clinical-radiographic disconnect, the weak outcome correlations mentioned in the Introduction confirm that anatomic bridging assessment—regardless of methodological sophistication—cannot reliably predict symptomatic outcomes.

These converging limitations explain the proliferation of assessment combinations [6,8]: attempts to address heterogeneity with increasingly specific categorical criteria paradoxically raise interpretive uncertainty. Clinicians remain unsure, sometimes confused, about which system best applies to their surgical context and patient presentation.

#### 4.2.3. Integrated Assessment Framework

Addressing these converging challenges requires a fundamentally different approach. Instead of pursuing increasingly detailed categorical criteria—which have led to the proliferation of numerous competing systems without reaching consensus [8]—this review introduces a principle-based interpretive framework built on two core principles.

(1) **Technique-factor correlation:** Bridging distribution should be assessed based on documented surgical variables—cage configuration (single versus double, footprint dimensions), graft placement location, and accessibility limitations—rather than applying universal thresholds uniformly across different surgical techniques. Expected bridging zones are derived from operative documentation for individual case, guided by the technical factors outlined in Section 3.2.2 [12,26,29].
(2) **Temporal-clinical integration:** Radiographic findings should be interpreted within the healing process and symptomatic context. Incomplete bridging at 12 months in an asymptomatic patient with stable hardware represents a different clinical entity than the same findings in a symptomatic patient with progressive lucency [21]—a distinction that categorical grading systems assigning definitive status at single timepoints cannot capture [4,14].

This framework shifts assessment from ‘Does this case meet predefined fusion criteria?’ to ‘Are the observed patterns consistent with expected healing given the documented surgical technique and clinical course?’ The framework does not eliminate the interpretive uncertainty inherent to fusion assessment; rather, it provides systematic principles for contextualizing radiographic findings. Section 4.3 describes how to operationalize this through standardized clinical workflows.

### 4.3. Practical Application of the Assessment Framework

**Figure 4:**
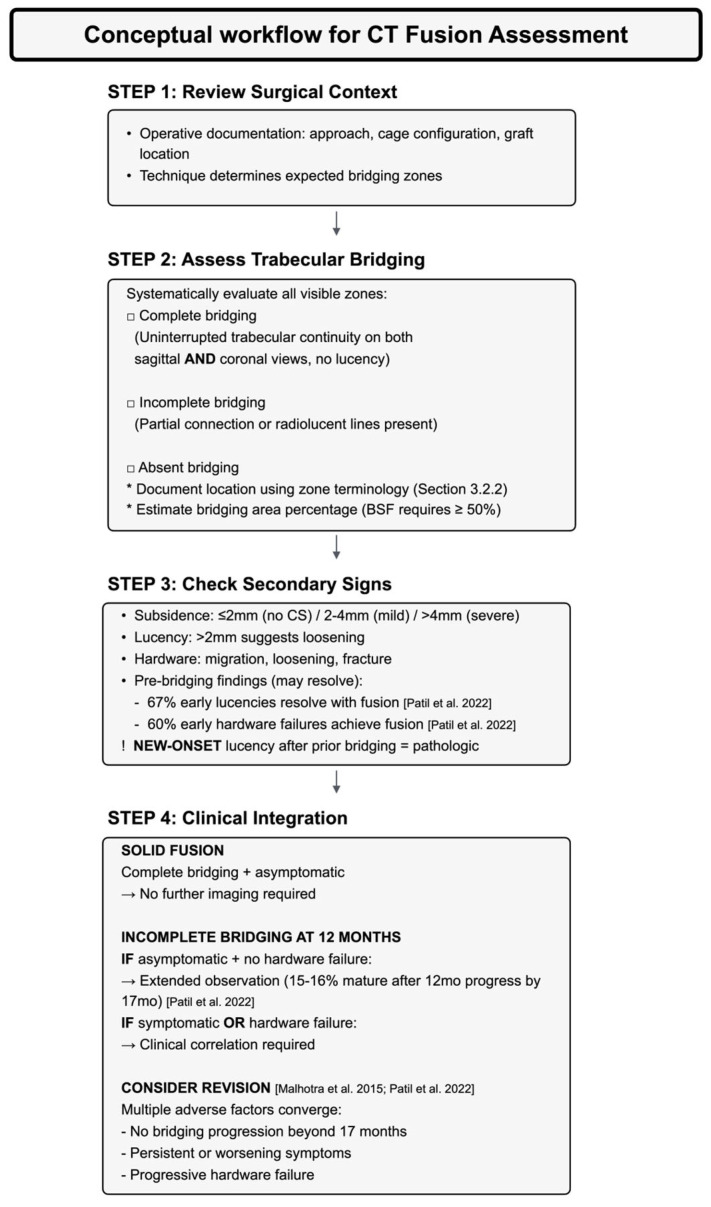
Conceptual workflow for CT fusion assessment. Sequential evaluation integrating surgical context (Step 1), trabecular bridging assessment (Step 2), secondary sign interpretation (Step 3), and clinical decision-making (Step 4). This framework distinguishes observation from intervention consideration based on radiographic findings, temporal healing patterns, and clinical presentation [15,21].

Clinical operationalization of the integrated assessment framework requires systematic implementation across three domains. (1) Image acquisition strategy involves optimizing protocols and selecting evidence-based timing to balance diagnostic benefits with radiation exposure. (2) Systematic interpretation and clinical decision-making integrate zone-based evaluation with surgical context, classification systems, and evidence-based revision thresholds, operationalized through structured algorithms (Figure 4, Table 1). (3) Standardized documentation guarantees reproducible communication via structured reporting templates, enabling longitudinal comparison and interdisciplinary collaboration.

#### 4.3.1. Image Acquisition Strategy

- Timing Selection

Clinical presentation determines imaging timing by applying the temporal healing patterns described in Section 3.1.5 to individual clinical scenarios. Contemporary practice prefers symptom-based assessment over protocol-driven assessment, reflecting technological advances and longitudinal outcome data.

For uncomplicated postoperative courses, assessment at 12 months provides adequate evaluation for most cases; cases lacking bridging bone beyond this time point warrant consideration of delayed fusion or pseudarthrosis [21]. This single-timepoint approach marks an evolution from traditional serial protocols that were necessary before artifact-reduction techniques enabled confident single-timepoint interpretation.

Earlier imaging (3–6 months) becomes necessary for clinical concerns—persistent pain, neurologic changes, or suspected hardware issues. However, interpretation should be done carefully: incomplete bridging at these timepoints usually reflects a physiological phenomenon of the normal healing process, with most early peri-implant lucencies resolving without intervention (Section 3.1.5) [21].

Extended follow-up to 17 months may be warranted when fusion status remains equivocal at 12 months, as fusion rates continue to improve substantially during this period [22]. This timing addresses the delayed maturation, with 15–16% of successful fusions still incomplete at 12 months. After 17 months, cases without bridging progression should be considered probable pseudoarthrosis and require clinical correlation [21].

This risk-stratified framework balances diagnostic timing with radiation exposure and intervention risk, recognizing that radiographic assessment offers probabilistic information that must be integrated with clinical context.

- Protocol Optimization

Standard fusion assessment employs thin-section acquisition (1–3 mm) with multiplanar reconstructions to visualize trabecular bridging. When hardware integrity becomes the main diagnostic concern—such as suspected screws loosening, cage migration, or fracture—dual-energy CT with high-keV monochromatic reconstructions (120–140 keV) enhances visualization of the hardware–bone interface [19]. The combined use of iterative reconstruction and monochromatic imaging provides synergistic metal-artifact reduction [20]. Importantly, parallel review of both artifact-reduced and conventional reconstructions remains essential, as overly aggressive artifact suppression may obscure true peri-implant lucency, which could indicate hardware loosening or early pseudarthrosis [18].

#### 4.3.2. Systematic Interpretation and Clinical Decision-Making

Systematic CT interpretation follows a step-by-step evaluation workflow that integrates surgical context, radiographic features, and clinical presentation (Figure 4). This framework incorporates evidence-based assessment criteria, temporal patterns, and decision thresholds (Table 1) to distinguish cases suitable for observation from those requiring additional intervention. The workflow consists of three sequential radiographic assessment steps (Steps 1–3), followed by clinical decision-making (Step 4).

- Surgical Context Integration

Interpretation starts with operative documentation—including surgical approach, cage configuration (single vs. double), graft material type and placement location, and technical variations—which provides context for bridging distribution (Figure 4, Step 1). Understanding how graft placement, cage geometry, and surgical accessibility influence expected bridging patterns (Section 3.2.2) allows for interpretation when findings deviate from expected configurations, such as significant bridging in ungrafted zones or reduced bridging in technically challenging regions despite proper graft placement [12,26].

- Trabecular Bridging Assessment

A systematic assessment of trabecular bridging across all visible zones involves using multiplanar sagittal and coronal reconstructions to categorize bridging as complete, incomplete, or absent (Figure 4, Step 2) [4,12,13]. Multiplanar verification is crucial because evaluating from a single plane can lead to an overestimation of bridging [12]. Documentation should specify the anatomical location using the zone terminology from Section 3.2.2 and estimate the percentage of the fusion area showing bridging, consistent with BSF criteria that require at least 50% bridging for radiographic fusion [4]. When bridging appears equivocal—for example, when continuity is visible on one plane but uncertain on orthogonal views—explicitly acknowledging this uncertainty helps guide appropriate follow-up actions [21].

- Secondary Sign Interpretation

Secondary sign evaluation uses diagnostic criteria and temporal patterns detailed in Table 1 (see also Figure 4, Step 3), distinguishing early findings that indicate normal healing from those suggesting complications. A key temporal distinction is that many early peri-implant lucency and hardware-related findings resolve with successful fusion, provided the instrumentation remains stable (Section 3.2.3). The clinical significance of new-onset versus early lucency is clarified in subsequent decision-making criteria.

- Temporal Progression Analysis

For patients with serial imaging, comparing images over time enhances the interpretation of secondary signs (Step 3) and guides subsequent clinical decisions (Step 4). Overall progression should be described as improving (more bridging, stable, or resolving secondary signs), stable, or deteriorating (no bridging growth but worsening secondary signs). Documentation should specify the recommended follow-up intervals and record meaningful changes observed during those times.

- Evidence-Based Observation Criteria

Interpretation of equivocal findings at the 12-month assessment integrates temporal healing patterns (Section 3.1.5 and Section 4.3.1) with clinical presentation (Figure 4, Step 4). Incomplete bridging at this time point might indicate delayed healing or early pseudarthrosis; distinguishing between them necessitates a multimodal evaluation.

Extended observation is reasonable when incomplete bridging occurs in asymptomatic patients with intact hardware (Figure 4), acknowledging that bridging may continue to mature beyond the standard assessment period [21,22]. This principle is exemplified by BSF type-2 fusion (locked pseudarthrosis), which shows favorable outcomes despite radiographic incompleteness, and hardware complications are absent once the bridging bone contacts both endplates [13,21]. Broader evidence indicates that symptomatic pseudarthrosis occurs in only 2.70% of multilevel fusion patients over 10 years, with symptoms appearing at a mean of 7.38 years, rates significantly lower than those of radiographic nonunion [37]. Although these data are not specific to BSF type-2 fusion, they support the concepts that radiographic incompleteness alone does not require intervention; clinical decisions should consider symptom status, hardware stability, and the progression over time.

Management of BSF type-2 fusion mainly relies on symptom correlation and construct configuration; comprehensive treatment algorithms go beyond this imaging-focused review. Early peri-implant lucency or hardware changes before bridging are considered in context over time, as these may indicate normal healing rather than failure (Section 3.1.5) [21]. When serial imaging is available, it further helps distinguish between normal and problematic findings by showing changes over time.

- Intervention Consideration Thresholds

Consideration of intervention is appropriate when multiple adverse factors converge (Figure 4). Symptomatic presentation—including persistent or worsening axial pain, new radiculopathy, or neurologic compromise—serves as the primary clinical indication. Radiographic criteria supporting the need for intervention include lack of bridging progression beyond 17 months, progressive hardware failure on serial imaging, or new-onset lucency after previously confirmed bridging. Clinical deterioration, manifesting as declining functional status or failure to meet expected postoperative milestones, provides additional indications for intervention consideration [21].

The distinction between temporary early findings and definitive failure remains essential. Hardware issues that occur before bridging formation may be due to variability in healing, whereas new lucency after established bridging strongly indicates hardware loosening and should be correlated clinically [21]. Combining clinical and radiographic information is essential; isolated CT findings—such as incomplete bridging, mild nonprogressive subsidence, or stable hardware—do not justify revision if there are no clinical signs of decline.

Operationalizing this interpretive framework requires structured documentation practices that ensure reproducibility, facilitate communication, and enable tracking over time (Section 4.3.3).

#### 4.3.3. Standardized Documentation

Standardized reporting operationalizes the assessment framework (Figure 4, Section 4.3.2) through systematic documentation. While institutional systems vary, comprehensive reports should include six essential components: procedure verification (treated levels, surgical approach, hardware configuration), primary assessment (bridging bone location and extent using anatomic zone terminology established in Section 3.2.2, overall fusion grade, explicit acknowledgment of equivocal findings), subsidence evaluation (cage position and severity grading: ≤2 mm no subsidence, 2–4 mm mild, or >4 mm severe [31]), hardware integrity assessment (peri-implant lucency > 2 mm suggesting loosening [3,5], complications including cage migration and screw loosening), clinical context (comparison with prior imaging, integration with expected healing timeline, symptomatic correlation), and identification of unexpected findings that require clinical correlation. Table 2 provides a condensed reporting checklist.

Documentation detail should reflect case complexity. Straightforward cases—such as clear fusion or definitive pseudarthrosis—require concise reporting, while equivocal cases warrant detailed zone-specific documentation, explicit acknowledgment of uncertainty, and recommended follow-up intervals [21]. Reports must distinguish between delayed consolidation and actual arrest, as uncertainty at a single time point does not necessarily indicate treatment failure. Documentation should focus on serial imaging and clinical correlation rather than premature conclusions that could lead to unnecessary intervention.

### 4.4. Limitation

#### 4.4.1. Biological and Clinical Complexities

Fusion assessment faces inherent biological complexities that influence all evaluation methods. Osseous healing is a biological continuum rather than distinct states, creating classification uncertainty, especially in locked pseudarthrosis (BSF type-2 fusion)—biomechanically stable but radiographically incomplete [13,14]. Incomplete bridging at 12 months may develop into solid arthrodesis, complicating single-timepoint categorical assessment [21]. These temporal patterns require serial imaging and recognition of interpretive uncertainty during intermediate healing phases.

Furthermore, CT criteria assess trabecular bridging patterns but cannot directly capture surgeon-dependent technical factors—including endplate preparation quality and cage positioning technique—or biologic adjunct composition (autograft, allograft, demineralized bone matrix, bone morphogenetic protein), which influence expected healing trajectories. These variables must be integrated through correlation with operative documentation rather than imaging assessment alone [3,5].

A well-documented disconnect between clinical outcomes and radiographic findings limits the usefulness of imaging for predicting results. While CT scans have high sensitivity for detecting pseudarthrosis (Section 4.2.1), a positive predictive value below 25% indicates that most radiographically incomplete fusions remain clinically stable [4]. Conversely, a solid radiographic fusion does not guarantee symptom relief, as there is a poor correlation between fusion grade and patient-reported outcomes [14,38]. This disconnect highlights that CT primarily assesses anatomical bridging rather than biomechanical stability or pain mechanisms. Clinical outcomes depend on factors beyond the fusion status, such as adjacent segment degeneration and residual neural compression—domains that require clinical correlation beyond radiographic imaging [39,40].

These biological and clinical complexities highlight the importance of integrating radiographic findings with temporal progression patterns and clinical presentation, as detailed in the systematic framework (Section 4.3), rather than relying solely on imaging criteria for clinical decision-making.

#### 4.4.2. Evidence Limitations

Contemporary fusion assessment relies on evidence that has significant methodological limitations. First, foundational validation studies establishing CT diagnostic accuracy relied on surgical exploration before 2010, which predates the widespread use of modern cage technologies and surgical techniques [4]. The BSF classification was created for radiolucent cages, but metallic implant artifacts can obscure intracage assessment, leading to lower inter-observer agreement [5,13,16].

Second, zone-specific reliability and fusion rate data derive primarily from TLIF, including prospective inter-observer assessment [12] and retrospective fusion rate analyses [26], which limits the extent to which findings can be applied to other interbody techniques. Data for ALIF-specific prospective reliability across assessment zones come from a single comparative imaging study with a much smaller sample size (14 patients, 25 levels) than the corresponding TLIF validation groups [41]. Studies comparing approaches show significant differences in fusion outcomes at the challenging lumbosacral junction (solid fusion: ALIF 75% vs. TLIF 47.9%, *p* = 0.006), with factors such as cage size, endplate preparation, and facet preservation affecting bridging patterns [24]. These studies do not thoroughly report variations in surgical technique, such as details about graft placement, endplate preparation, cage geometry, or surgical access, which are important for understanding bridging patterns. This lack of detailed documentation makes it difficult to link technical factors directly to radiographic results and contributes to the high variability observed in current research [8]. A similar systematic review of posterolateral fusion shows comparable issues—with 47% using classification systems, 63% using descriptive criteria, and only 55–80% agreement between imaging and surgical findings—highlighting that inconsistent criteria are a common problem across different fusion methods [42]. Therefore, validating the overall assessment framework (Section 4.2.3 and Section 4.3) across different surgeries and cage designs remains a significant challenge.

Future multi-center prospective validation should standardize documentation of technique-specific variables: cage configuration (size, footprint, material composition), graft material composition and placement, extent of endplate preparation, and surgical accessibility limitations. This systematic reporting will enable correlations between surgical details and zone-specific bridging patterns, thereby furthering evidence-based fusion evaluation.

#### 4.4.3. Review-Specific Limitations

This narrative review has several inherent limitations. First, the search strategy was limited to English-language publications indexed in PubMed and Google Scholar, which may have excluded relevant studies in other languages or databases. Second, since it is a narrative rather than a systematic review, study selection was based on the author’s discretion without a formal quality assessment or risk-of-bias evaluation. Third, the scope was intentionally restricted to CT-based fusion assessment, excluding magnetic resonance imaging, dynamic radiography, and emerging modalities that could offer additional diagnostic information. Finally, the review focused solely on lumbar interbody fusion, so the findings may not be applicable to posterolateral fusion techniques or cervical/thoracic spine procedures.

Despite these constraints, this review synthesizes current evidence to offer practical guidance for modern CT-based fusion assessment. The limitations underscore the importance of integrating radiographic findings with clinical presentation and temporal progression patterns, as emphasized throughout Section 4.2 and Section 4.3, rather than relying solely on imaging criteria.

### 4.5. Future Directions

#### 4.5.1. Standardization of Documentation and Interpretive Principles

The heterogeneity in fusion assessment methodologies—reflecting substantial methodological variation [8]—highlights the need for systematic documentation practices. Future efforts should focus on standardizing two key areas: surgical technique documentation and radiographic interpretation frameworks.

Standardized reporting of surgical variables requires multidisciplinary collaboration to define key technical parameters, including cage configuration, graft composition and placement distribution, extent of endplate preparation, and surgical accessibility constraints. Such documentation would allow investigation of how multiple interacting technical factors affect bridging patterns and healing trajectories.

Regarding outcome categorization, recognizing that osseous healing exists on a biological spectrum, clinical decisions may benefit from a simple classification (observation-appropriate versus intervention-consideration) for practical management, while detailed multi-grade documentation supports research and quality monitoring [43]. This complementary approach balances clinical usefulness with research accuracy.

#### 4.5.2. Clinical-Radiographic Correlation and Outcomes Research

Despite extensive focus on radiographic fusion criteria, the relationship between CT-based interbody fusion assessment and patient-reported outcomes remains a critical knowledge gap. Limited evidence from posterior instrumented fusion in adult spinal deformity suggests that radiographic fusion grade does not significantly impact health-related quality of life in the absence of hardware failure, with instrumentation integrity proving more predictive of clinical deterioration than fusion status alone [38]. Preliminary data from minimally invasive interbody fusion similarly show no significant correlation between radiographic fusion grade and patient-reported outcomes [14]. Long-term registry data confirm comparable clinical outcomes between anterior and posterior approaches at 8-year follow-up despite known technique-specific variations in fusion patterns [44]. However, direct validation linking specific radiographic patterns with clinical outcomes has not been systematically addressed, representing a significant research priority given the distinct biomechanical and anatomical characteristics of cage-based fusion constructs.

Prospective cohort studies should assess which specific radiographic patterns are associated with clinical stability versus symptomatic pseudarthrosis that requires additional intervention. Key research questions include: Do specific zone-specific bridging patterns predict mechanical stability? Does the pattern of temporal progression affect long-term outcomes? Which secondary radiographic signs show meaningful clinical associations with persistent symptoms?

Investigation should also focus on the optimal imaging surveillance strategy: determining which patients benefit from serial CT monitoring rather than clinical assessment alone, and identifying radiographic patterns that warrant extended observation intervals. Such research would shift fusion assessment from merely documenting anatomical healing to predicting clinically relevant outcomes.

#### 4.5.3. Artificial Intelligence and Quantitative Analysis

Addressing the research priorities outlined above will require efficient, reproducible assessment tools. AI-based tools offer potential to improve reproducibility and efficiency in fusion assessment [45,46,47]. Current applications in spinal imaging include automated vertebral segmentation, parameter measurement, and degenerative change detection, with demonstrated accuracy for automated sagittal balance analysis, achieving intraclass correlation coefficients of 0.71–0.99 compared to manual measurements [46,48,49]. Extending this approach to fusion assessment could help with time-consuming tasks such as multi-zone bridging evaluation and tracking changes over time. Machine learning algorithms could potentially automate zone-specific bridging quantification and identify subtle temporal changes across serial exams [47]. This automation would be especially useful for the multi-zone assessment approach described in Section 3, where a thorough evaluation of anterior, lateral, posterior, and intracage regions requires systematic review that might benefit from computational assistance.

However, successful clinical integration of AI requires addressing key challenges. Training datasets must represent the technical diversity outlined in Section 3.2.2, covering various cage designs and surgical methods. Validation should evaluate not only diagnostic accuracy but also calibration across different time points, since interpretation principles differ between early post-operative and late assessments. Future work should define how AI-generated assessments support, rather than replace, radiologists’ interpretation, especially for cases where secondary signs and their temporal changes are important.

#### 4.5.4. Advanced Imaging Techniques

Photon-counting CT is a promising technological advancement, offering reduced electronic noise and enhanced tissue contrast compared to conventional energy-integrating detectors [34,50]. Quantitative analysis shows significantly improved signal-to-noise and contrast-to-noise ratios for bone imaging under standardized conditions, although subjective visualization of trabecular structures remains similar between detector types when imaging parameters are matched [51]. Early spine studies indicate effective metal artifact reduction with virtual monoenergetic imaging and suggest the potential for lower radiation doses [52].

These quantitative benefits may offer objective metrics that complement categorical bridging assessments, especially for tracking changes over time. Importantly, virtual monoenergetic imaging at higher keV levels seems crucial for effective metal artifact reduction with photon-counting detectors [51,52]. However, clinical use requires spine fusion-specific validation linking these imaging biomarkers with fusion outcomes, along with practical considerations of cost and availability in routine practice settings.

## 5. Conclusions

CT remains the reference standard for assessing LIF. However, documented methodological variability—due to technique-specific differences, the biological healing process, and a disconnect between clinical and radiographic findings—shows that strict categorical approaches are inadequate for modern practice. This review emphasizes that dependable fusion assessment requires systematic integration of three key elements: technical optimization, principle-based interpretation, and temporal–clinical context.

Modern CT protocols that incorporate iterative metal artifact reduction and dual-energy imaging significantly improve visualization of the hardware–bone interface, providing a solid technical foundation for systematic, zone-based evaluations. Validation studies show that bridging patterns depend on technique-specific factors such as cage configuration, graft placement, and surgical accessibility, rather than on the surgical approach itself. A comprehensive interpretation integrates the BSF classification with zone-specific documentation, linking radiographic findings to details of the surgical procedure to support individualized assessments.

Evidence-based temporal context guides clinical decision-making: routine assessment at 12 months yields the optimal diagnostic window, while a subset of successful fusions shows delayed progression to solid arthrodesis by 17 months. Intervention consideration requires the convergence of multiple adverse factors—persistent symptoms, progressive hardware failure, and arrested bridging progression—rather than relying on isolated radiographic findings.

Fundamental limitations remain: CT assesses anatomical bridging but cannot reliably predict biomechanical stability or symptom relief. This intrinsic disconnect between clinical and radiographic findings requires careful clinical correlation for all management decisions. Future research should focus on prospectively validating zone-specific criteria across different cage designs, graft strategies, and approach-related limitations, while exploring quantitative techniques to improve the objectivity of assessment.

## Figures and Tables

**Table 1:** Secondary Radiographic Signs: Diagnostic Criteria and Clinical Implications.

	Threshold/Grade	Diagnostic Criteria	Clinical Significance
**Cage Subsidence**			
Non-significant	<2 mm DH loss	Absolute measurement	Normal implant settling without clinical significance [31]
Mild	2–4 mm DH loss	Absolute measurement	Early phenomenon; typically stabilizes by 3 months; minimal long-term fusion impact [31]
Severe	>4 mm DH loss	Absolute measurement	Clinical significance varies by construct type and fixation strategy; requires correlation with symptoms and serial imaging [31]
**Cage Migration**	Directional assessment	Posterior or lateral (neural foramina), anterior (vascular risk)	Significance depends on symptom correlation; asymptomatic cases may require observation [27,34]
**Endplate-Cage Interface Changes**	Qualitative (serial imaging)	Cystic changes at cage-endplate interface	Indicates inadequate stability and delayed fusion; resolution on follow-up suggests fusion progression [3]
**Preoperative** **Bone Quality (HU)**			
Adequate	>127–142 HU	Level-specific thresholds: L4 > 127, L5 > 136, S1 > 142 HU	Associated with fusion success [32]
Compromised	≤127–142 HU	Below level-specific thresholds	Increased pseudarthrosis risk; low bone quality associated with subsidence [31,32]
**Hardware Integrity**			
Peri-implant lucency	>2 mm	Progressive lucent zone on serial CT	Suggests implant loosening; ~67% early lucency resolves with successful fusion [3,5,18,21]
Hardware fracture	Visible discontinuity	Hardware failure on CT	Assess for pseudarthrosis; ~60% achieve fusion if the segment remains stable [15,21]

Abbreviations: DH, disc height; HU, Hounsfield unit.

**Table 2:** Minimum Reporting Elements for CT Fusion Assessment.

Component	Essential Elements
1. Surgical Context	Time from surgery, treated levels, surgical approach, cage configuration (single vs. double, footprint dimensions), and graft material and placement location
2. Trabecular Bridging Assessment	Bridging status by zone (complete/incomplete/absent), estimated percentage of bridging area, BSF grade, explicit acknowledgment of equivocal findings
3. Secondary Signs	Cage subsidence (<2 mm/2–4 mm/>4 mm), cage migration, endplate-cage interface changes, peri-implant lucency (>2 mm suggests loosening), and hardware integrity
4. Clinical Context	Comparison with prior imaging, correlation with expected healing timeline, and symptomatic status
5. Follow-up Recommendation	Routine follow-up/Extended observation/Clinical correlation advised/Further evaluation recommended

Abbreviations: BSF, Brantigan-Steffee-Fraser classification. Subsidence and lucency thresholds referenced from Table 1.

## Data Availability

No new data were created or analyzed in this study. Data sharing is not applicable to this article.

## Abbreviations

The following abbreviations are used in this manuscript:

BSF	Brantigan-Steffee-Fraser classification
AI	Artificial intelligence
CT	Computed tomography
DECT	Dual-energy computed tomography
DH	Disc height
FBP	Filtered back projection
HU	Hounsfield unit
ICC	Intraclass correlation coefficient
IMAR	Iterative metal artifact reduction
KeV	Kiloelectron volt
KVp	Kilovolt peak
LIF	Lumbar interbody fusion
ALIF	Anterior lumbar interbody fusion
LLIF	Lateral lumbar interbody fusion
OLIF	Oblique lumbar interbody fusion
PLIF	Posterior lumbar interbody fusion
TLIF	Transforaminal lumbar interbody fusion
PEEK	Polyetheretherketone
